# Gelatin-Functionalized Carbon Nanotubes Loaded with Cisplatin for Anti-Cancer Therapy

**DOI:** 10.3390/polym15163333

**Published:** 2023-08-08

**Authors:** Rong Li, Zhenfei Bao, Pei Wang, Yunyun Deng, Junping Fan, Xin Zhu, Xinyu Xia, Yiming Song, Haiyan Yao, Dongfang Li

**Affiliations:** 1 School of Stomatology, Nanchang University, Nanchang 330006, China; lirong012@email.ncu.edu.cn (R.L.); baobaobaozhenfei@163.com (Z.B.); ndfskqyy620@ncu.edu.cn (P.W.); ndfskqyy622@ncu.edu.cn (Y.D.); jpfan@email.ncu.edu.cn (J.F.); zxaiyf99@163.com (X.Z.); xiaxinyu202306@163.com (X.X.); songyiming963@163.com (Y.S.); ncuyaohaiyan@163.com (H.Y.); 2 The Key Laboratory of Oral Biomedicine, Jiangxi Province, Nanchang 330006, China; 3 Jiangxi Province Clinical Research Center for Oral Diseases, Nanchang 330006, China

**Keywords:** carbon nanotubes, cisplatin, drug delivery systems, biocompatibility, release

## Abstract

Cisplatin (Cp), a chemotherapeutic agent, interacts with purines on tumor DNA, causing tumor cell apoptosis. However, cisplatin has the characteristics of non-specific distribution and lack of selectivity, resulting in systemic toxicity. Moreover, it cannot maintain the drug’s high concentration in the tumor-weak acid environment. These flaws of cisplatin restrict its use in clinical applications. Therefore, a pH-responsive carbon nanotube-modified nano-drug delivery system (CNTs/Gel/Cp) was constructed in this study using gelatin (Gel)-modified carbon nanotubes (CNTs/Gel) loaded with cisplatin to release drugs precisely and slowly, preventing premature inactivation and maintaining an effective concentration. When M_Cp_:M_CNTs/Gel_ = 1:1, the drug reaches the highest loading rate and entrapment efficiency. To achieve the sustained-release effect, CNTs/Gel/Cp can release the medicine steadily for a long time in a pH environment of 6.0. Additionally, CNTs/Gel/Cp display antitumor properties comparable to cisplatin in a manner that varies with the dosage administered. These findings indicate that CNTs/Gel/Cp have an effective, sustained release of cisplatin and a good antitumor effect, providing a theoretical and experimental basis for the clinical application of modified carbon nanotubes (CNTs) as a new drug delivery system.

## 1. Introduction

Cancer prevalence escalates annually and remains among the primary causes of human mortality globally [1]. Amongst them, oral cancer stands as the sixth most prevalent one, posing a significant public health challenge [2]. Surgical intervention for oral cancer may engender facial disfigurement, while both surgical and radiotherapeutic approaches may elicit significant functional side effects, most notably impinging upon a person’s abilities to ingest food, drink, and communicate verbally. By fostering the development of innovative chemotherapy agents and effective drug delivery systems that enhance the anti-neoplastic effects of drugs, the survival rates, and quality of life for those affected by oral cancer may be elevated [3]. In clinical oncology, chemotherapy is still a vital treatment option. Cisplatin (Cp), a well-known and broad-spectrum anticancer medication, is regarded as one of the most effective chemotherapy agents now used in clinical practice [4]. As a low molecular weight platinum compound with high reactivity and strong accumulation properties [5], cisplatin is irreversibly bound to plasma proteins upon administration, leading to its low bioavailability. Therefore, less than 10% of cisplatin can exert its antitumor effects freely [6]. Hence, this chemotherapeutic agent’s class has issues, such as poor bioavailability, specificity, and substantial toxic side effects [7]. Evidence indicates that utilizing nano-delivery systems as transport vehicles for chemotherapy drugs enables a gradual and controlled drug release at the tumor site, thus improving their therapeutic efficacy and minimizing chemotherapy-related adverse effects [8,9]. Innovative cancer treatment, utilizing nano-delivery systems to provide chemotherapy medications, is an effective technique that avoids the limits of traditional delivery systems, including drug inactivation and resistance [10,11,12].

Currently, research on nanoparticle drug delivery systems has made significant progress [13,14], with a focus on stimuli-responsive controlled-release systems like that of pH-responsive [15], redox-responsive [16], photoresponsive [17], and multi-responsive systems [18,19]. Among them, the pH-responsive drug delivery system exploits the pH difference between tumor microenvironments and normal cells to achieve effective drug release control without external stimuli [20]. Carbon nanotubes (CNTs) are a classic carbon-based nanomaterial essential for nano-drug delivery systems due to their high drug-loading capacity, robust adsorption capabilities, and high specific surface area. Their high drug-loading capacity and excellent ability to penetrate cells have also piqued the interest of many researchers [21,22]. Due to carbon nanotubes’ single- or multi-walled nature, proteins can be encapsulated within them to produce sustained and targeted drug delivery [23]. Additionally, the needle-like structure of carbon nanotubes enables them to enter cells, aiding effective medication delivery and specific killing of tumor cells [24,25]. By possessing this ability, drugs can avoid premature inactivation, minimize damage to neighboring healthy cells, and postpone the emergence of chemoresistance [10,26,27].

However, carbon nanotubes’ possible toxicity and hydrophobicity have hampered their application in nano-drug delivery [28,29,30]. Carbon nanotubes’ self-aggregation phenomenon can impact biomolecule encapsulation [31,32]. Therefore, carbon nanotubes must be functionalized before effectively being used in cancer treatment [33,34]. Appropriately functionalizing the carbon nanotubes’ surface can greatly enhance their performance and biocompatibility. Functionalized carbon nanotubes can enhance the cellular uptake of low-permeability drugs, thereby increasing their intracellular concentration and improving their efficacy [35]. Functionalizing the carbon nanotubes’ surface can also ameliorate chemotherapy-induced drug resistance while further improving the carbon nanotubes’ loading capacity [26,32,36]. We created a polydopamine (PDA) coating by self-polymerizing dopamine (DA) [37], comprising reactive groups that permit Michael’s addition reactions with gelatin (Gel) molecules. Dopamine possesses a strong drug adsorption capability, enhancing the carrier system’s drug-loading capacity [38,39]. Gelatin can make carbon nanotubes more soluble and less harmful to biological systems [40]. We enhanced carbon nanotubes’ biocompatibility and dispersion by functionalizing their surface with polydopamine and gelatin. Our preliminary research findings [41] show that, compared to pristine multi-walled carbon nanotubes (MWCNTs), CNTs/Gel exhibit higher water solubility, lower cytotoxicity, significantly increasing biocompatibility, and resolving carbon nanotube aggregation and toxicity issues. Doxorubicin [33], cisplatin [42], paclitaxel [43], and camptothecin [44] are now used as nano-delivery systems for chemotherapy drugs based on carbon nanotubes. These can be grafted onto carbon nanotubes by physical loading, covalent, non-covalent, and redox reactions [45]. Additional research suggests that surface-modified carbon nanotubes can substantially improve drug concentrations in the bloodstream, thus improving the antitumor effects of therapy [46].

**Figure 1:**
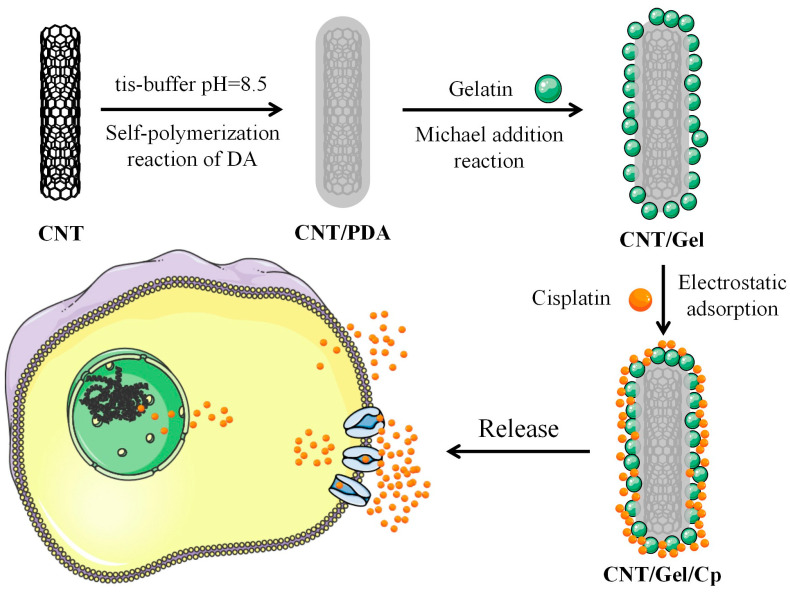
Illustration of the process for the preparation of CNT/Gel/Cp and its mechanism of action for antitumor effects.

Using previously manufactured gelatin-surface-modified carbon nanotubes (CNTs/Gel) as the drug delivery vehicle for cisplatin loading, we created a pH-responsive nano-drug delivery system in this study (Figure 1). As an emerging drug delivery system, modified CNTs were evaluated for their drug-loading capacity, encapsulation effectiveness, and release properties, and preliminary research was conducted on their anticancer effect in vitro. This evaluation was a fundamental guide for the clinical application of modified CNTs. The null hypotheses are: (1) CNTs/Gel/Cp cannot achieve a controlled release of Cp drugs; (2) CNTs/Gel/Cp do not exhibit anticancer effects in the slightly acidic tumor microenvironment.

## 2. Materials and Methods

### 2.1. Materials

CNTs were bought from Xianfeng Nanomaterial Technology Co., Ltd. (Nanjing, China), and Tris and gelatin were purchased from Sinopharm Chemical Reagent Co., Ltd. (Shanghai, China). Cisplatin was purchased from BASF Biotech Co., Ltd. (Anhui, China). Squamous cell cal-27 was bought from the Shanghai Cell Bank of the Chinese Academy of Sciences, and the Cell Counting Kit-8 was purchased from Aladdin Biochemical Technology Co., Ltd. (Nanjing, China).

### 2.2. Surface Modification of CNTs with PDA

CNTs were modified by PDA following the prior experimental procedure to use them as carriers [41]. First, the CNTs (0.25 g) were distributed into a 250 mL tris-buffer solution (pH 8.5), stirring for 30 min. Then, dopamine (250 mg) was incorporated into the above solution and stirred for 4 h. Finally, CNTs/PDA was gathered through centrifugation and washed thrice with deionized water.

### 2.3. Modified CNTs with Gelatin (CNTs/Gel) Preparation

The CNTs/PDA surface was grafted with gelatin by the Micheal addition reaction. First, CNTs/PDA, obtained by the method described above (Section 2.2), were dispersed in 100 mL of water and stirred for 30 min. Then, dissolving 0.5 g of gelatin in 250 mL of deionized water took place, and then the above two solutions were mixed, stirring for 24 h. Finally, the CNTs/Gel product was collected by centrifugation, washed thrice with deionized water, and freeze-dried for 24 h.

### 2.4. CNTs/Gel/Cp Preparation

To make a suspension, we mixed the 10 mg product (CNTs/Gel) from Section 2.3 equally with 20 mL of water. Then, another 10 mg of cisplatin was added to the suspension. After 12 h of stirring and then centrifuging, the supernatant was removed. Finally, the product (CNTs/Gel/Cp) was collected by lyophilizing for 24 h.

### 2.5. Characterizations

#### 2.5.1. Scanning Electron Microscope (SEM)

The microscopic morphology of the CNTs/Gel and CNTs/Gel/Cp samples was monitored by a scanning electron microscope (SMZ800, Nikon, Tokyo, Japan). The unfixed powder was eliminated by fixing the freeze-dried samples to the sample stage with conductive tape. After that, the samples’ surface morphologies were examined at a 15 kV voltage.

#### 2.5.2. TEM and EDS

A transmission electron microscope (JEM-F200, JEOL, Tokyo, Japan) operating at 200 kV was used to observe the microstructure of the CNTs/Gel and CNTs/Gel/Cp samples. The samples’ chemical compositions were determined using EDS to identify the types, quantities, and distribution of the chemical elements present.

#### 2.5.3. Dynamic Light Scattering (DLS)

A laser particle sizer (Zetasizer nano zs90, Malvern, UK) was used in the dynamic light scattering (DLS) system to measure the average diameter and size distribution of the CNTs/Gel and CNTs/Gel/Cp samples. All analyses were repeated in triplicate, and the data represented the mean of three measurements.

#### 2.5.4. Zeta Potential

The Zeta potential of the prepared samples was measured by a laser particle size analyzer (Zetasizer nano zs90, Malvern, UK) of a dynamic light scattering (DLS) system. It symbolizes a potential variance between the dispersion medium and CNTs/Gel/Cp. All analyses were repeated thrice, and the data were the average of three measurements.

#### 2.5.5. Fourier Transform Infrared (FITR) Spectroscopy

Infrared spectroscopy (Nicolet 6700, Thermo Fisher Scientific, Waltham, MA, USA) examined the functional groups and chemical structures of the Cp and CNTs/Gel/Cp samples. After the above two groups of powders were combined with KBr particles, respectively, FTIR measurements were conducted in the infrared range (4000–500 cm^−1^) with a resolution of 2 cm^−1^. Briefly, 128 scans were completed using an infrared spectrometer at room temperature.

#### 2.5.6. Ultraviolet Spectroscopy

The spectral analyses of Cp and CNTs/Gel/Cp were performed using an ultraviolet spectrophotometer (UV-5200, Shanghai, China). Cp group: 10 mg of Cp was uniformly dissolved in 100 mL of water to create a dispersed suspension—at room temperature, 106 scans were performed. CNTs/Gel/Cp Group: A dispersed suspension of 10 mg of CNTs/Gel/Cp was prepared by equally distributing it into 20 mL of water—scanning was conducted 106 times at room temperature.

#### 2.5.7. X-ray Diffraction (XRD) Spectra and Raman Spectroscopy

X-ray diffraction (XRD) spectra are non-destructive techniques used to identify crystal phases and analyze the structural characteristics of solid materials. The CNTs/PDA, CNT/Gel, and CNTs/Gel/Cp spectra were obtained at room temperature using a Japanese Rigaku SmartLab SE diffractometer with Cu-Kα radiation at a working voltage of 40 kV over a scanning range of 2θ = 5–90°.

Using a Raman spectrometer (LabRAM HR Evolution, Horiba, Kyoto, Japan), the surface and interface properties of CNTs and their derivatives were studied by analyzing the shift of characteristic peaks and obtaining insights into their surface modification and reactivity. The excitation laser had a wavelength of 532 nm.

#### 2.5.8. TGA Analysis

The degree of functionalization and thermal stability of CNTs was verified using TG-DSC analysis. TG-DSC data were obtained using a thermal analyzer (STA 6000, PerkinElmer, Waltham, MA, USA) under a nitrogen atmosphere and were used to generate a TGA curve. The test temperature range was 30–500 °C with a heating rate of 10 °C/min.

### 2.6. Drug Loading

Three groups of M_Cp_:M_CNTs/Gel_ = 1:2, 1:1, 2:1 solutions were prepared. First, 10 mg CNTs/Gel was added to 20 mL of water in each group, and ultrasonic shock was conducted for 2–4 min to make it evenly dispersed. Afterward, each group received 5, 10, and 20 mg of Cp, respectively, and were stirred for 12 h (450 rpm) at room temperature. Samples were collected by centrifugation (8000 rpm, 12 min), and the supernatant was extracted. Finally, using an ultraviolet spectrophotometer to measure the supernatant’s absorbance at 204 nm, its value was calculated using the standard curve calculation formula:

Encapsulation efficiency = (M_cp_ − M_solution_)/M_cp_ × 100% (1)

Loading rate = (M_cp_ − M_solution_)/(M_cp_ + M_CNTs/Gel_) × 100% (2)

M_CNTs/Gel_ (mg) represents the mass of the CNTs/Gel incorporated as carriers in each group.

M_solution_ (mg) represents the mass of cisplatin, calculated from the standard curve in the supernatant of each group.

M_cp_ (mg) represents the total mass of cisplatin added in each group, which is equivalent to the mass of cisplatin loaded on CNTs/Gel plus M_solution_ (mg), indicating the mass of cisplatin in the supernatant.

### 2.7. In Vitro Release Studies

According to the above method (Section 2.6), the CNTs/Gel/Cp group with the highest load rate was selected, and the CNTs/Gel/Cp group was divided into two groups (n = 3) according to the different pH values. The experiment was repeated thrice in each group, and 3 mL of PBS solution (pH = 7.4 and pH = 6.0) was added to each group, respectively. The above solution was placed into a dialysis bag (molecular cut-off 4000)—sealing at both ends—and placed in 50 mL of PBS solution (pH = 7.4 and pH = 6.0). The samples were released continuously and steadily with a shaker at 37 °C. After obtaining 3 mL samples at various times, the overall volume of the solution was maintained constant by adding the same amount of buffer to the original solution. The Cp release in the samples was measured by an ultraviolet spectrophotometer at 204 nm. Each sample was tested thrice.

### 2.8. In Vitro Antitumor Effect

The human tongue squamous cell carcinoma cell Cal-27 viability was determined by the CCK-8 method. Human tongue squamous cell carcinoma cells Cal-27 were cultured and seeded in a 96-well plate for 24 h according to a certain number (6 × 10^3^ cells/well), and each well contained 100 μL of cancer cell fluid. Cancer cells were treated with Cp and CNTs/Gel/Cp at concentrations of 1, 2, 5, 10, and 20 μg/mL, respectively, establishing five repeat wells and incubating for 24 h. After culturing for 24 h, each well received 10 mL of CCK-8 reagent and was incubated for another 2 h in the incubator. Using a microplate reader, the absorbance value was measured, and cell viability was checked in each well of the sample at a 450 nm wavelength.

The antitumor effect was evaluated by using the AO/EB staining method. The human tongue squamous cell carcinoma cell Cal-27 was seeded and cultured at a density of 6 × 10^3^ cells/well in a 96-well plate containing 1 mL of 10% FBS high-glucose DMEM medium for 24 h. After adding 1 mL of the new medium containing the Cp and CNTs/Gel/Cp at concentrations of 1, 2, 5, 10, and 20 μg/mL, the medium was changed. Cancer cells were then incubated with Cp and CNTs/Gel/Cp for 24 h while the fresh medium was added, and the plate holes were cleaned multiple times. Finally, after adding the AO/EB reagent for 30 s, the sample was observed by fluorescence microscope (Zeiss, AXIO Observer Z1, Jena, Germany).

## 3. Results and Discussion

### 3.1. Characterizations

#### 3.1.1. Scanning Electron Microscopy (SEM)

**Figure 2:**
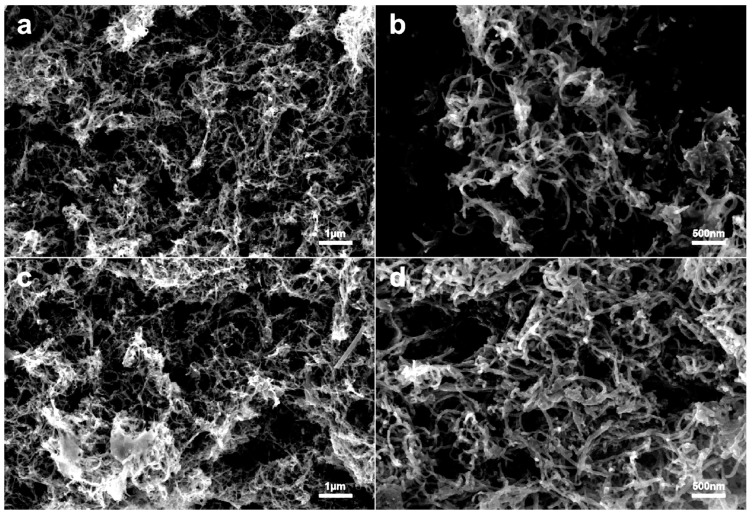
Representative SEM images (10,000×) are shown in the left column: (**a**) CNTs/Gel group, (**c**) CNTs/Gel/Cp group. The higher enlargement images (20,000×) of the respective groups are shown in the right column (**b**,**d**). The volume and diameter of the nanotubes significantly increase, and their surface becomes rougher.

At 10,000× magnification, the scanning electron microscopy (SEM) analysis revealed that CNTs/Gel exhibited a network topology of intertwined nanotubes with excellent dispersion and a relatively smooth surface (Figure 2a). At a magnification of 20,000 times, the SEM image showed that the network structure of CNTs/Gel consisted of interconnected tubular structures, which were highly hydrophilic and well-dispersed (Figure 2b). Compared to CNTs/Gel (Figure 2a), CNTs/Gel/Cp demonstrated a comparable dispersion with a rougher surface. They visibly improved adsorption, considerably increasing the nanotubes’ volume and diameter (Figure 2c). The zoomed-in images confirmed that polyhedral-shaped cisplatin was absorbed into the network structure of CNTs/Gel/Cp (Figure 2d). The SEM results verified that the gelatin surface modification of CNTs significantly improved their surface properties, enabling superior dispersion and facilitating cisplatin loading, as reported in our previous study [41]. After adsorption, the resulting rough surface and variations in volume and diameter revealed the successful loading of cisplatin while preserving excellent nanotube dispersion.

#### 3.1.2. TEM and EDS

**Figure 3:**
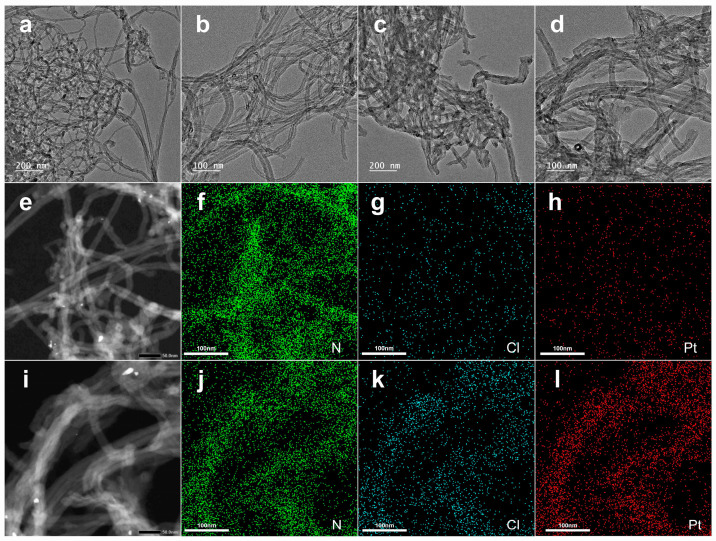
The first row presents the representative TEM images: (**a**) CNTs/Gel group, (**c**) CNTs/Gel/Cp group, bar = 200 nm. (**b**,**d**) Illustrate the high magnification TEM images for each group, bar = 100 nm. TEM observation revealed a rougher surface and uniform material adsorption for CNTs/Gel/Cp. (**e**) Dark-field image of CNTs/Gel. (**f**–**h**) Elemental mapping of (**e**). (**i**) Dark-field image of CNTs/Gel/Cp. (**j**–**l**) Elemental mapping of (**i**). Cisplatin was adsorbed onto the surface of carbon nanotubes and aligned along the long axis of the nanotubes. The Cl and Pt elements distribution in the CNTs/Gel/Cp group was significantly higher in element content than in the CNTs/Gel group.

Further observation of the sample’s morphology features was conducted through the transmission electron microscope. CNTs/Gel were equally disseminated in a mesh-like structure in the medium, with no discernible size fluctuations and a smooth surface (Figure 3a,b). In contrast, CNTs/Gel/Cp exhibited a rougher surface and uniform material adsorption. The mesh structure in CNTs/Gel/Cp became denser, with a noticeable increase in diameter size (Figure 3c,d). These pictures show that cisplatin was successfully electrostatically attracted to the surface of carbon nanotubes without changing their natural dispersion. Functionalization plays a critical role in preventing nanotube aggregation and ensuring a stable dispersion [15]. The EDS results added more proof to this conclusion.

The energy dispersive spectroscopy (EDS) analysis revealed that cisplatin was adsorbed onto the surface of carbon nanotubes and aligned along the long axis of the nanotubes. The Cl and Pt elements distribution in the CNTs/Gel/Cp group was consistent with the spatial distribution of carbon nanotubes, with a significantly higher element content than that in the CNTs/Gel group (Table 1), in agreement with the EDS analysis reported in the literature [47]. These findings demonstrated that cisplatin was successfully loaded onto carbon nanotubes (Figure 3e–i).

#### 3.1.3. DLS and Zeta Potential

**Figure 4:**
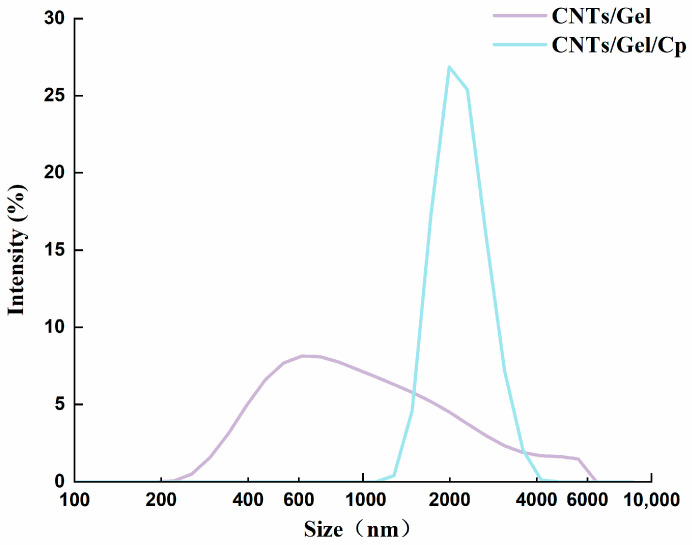
DLS of CNTs/Gel and CNTs/Gel/Cp. The average particle size of CNTs/Gel measures 848 nm, and CNTs/Gel/Cp measures 2147 nm.

The physicochemical characteristics of all the nanoparticles were further investigated by performing dynamic light scattering (DLS) studies on their particle size distribution. The average particle size of CNTs/Gel measures 848 nm, whereas that of CNTs/Gel/Cp measures 2147 nm (Figure 4). The rise in the average particle size of CNTs/Gel/CP strongly suggests that cisplatin was loaded successfully [15].

**Figure 5:**
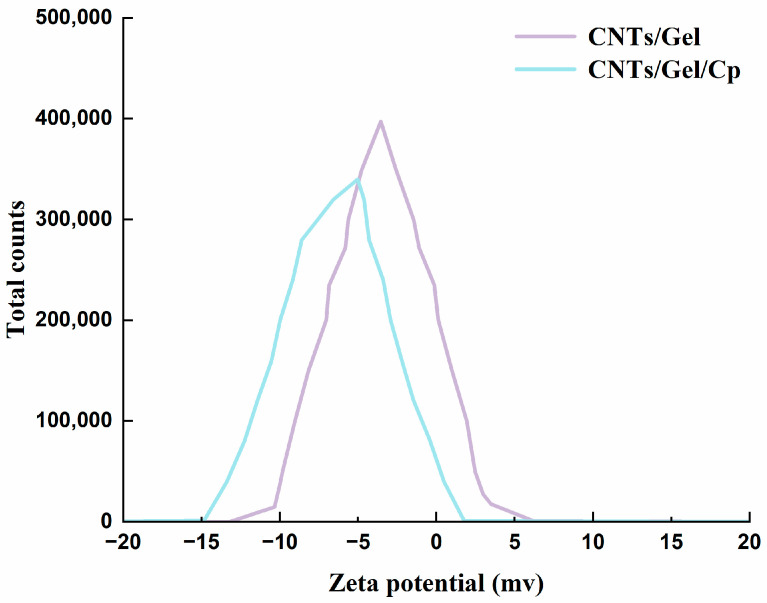
Zeta optional of CNTs/Gel and CNTs/Gel/Cp. The extremely negative value may be due to the functionalization of the nanoparticles, which increases the negative charge on their surface.

In Figure 5, the negative Zeta potential of CNTs/Gel is likely due to the carboxyl and hydroxyl functional groups on its surface. The negative charge contributes to the electrostatic repulsion between negatively charged clusters, thereby discouraging aggregation and promoting the stability of CNTs/Gel [48]. CNTs/Gel/Cp has a smaller negative Zeta potential than CNTs/Gel (Figure 5), and its extremely negative value may be due to the functionalization of the nanoparticles, which increases the negative charge on their surface. Further, the cisplatin addition allows for a stable dispersion of CNTs/Gel/Cp in solutions close to the Zeta potential, resulting in the partial ionization of cisplatin and CNTs/Gel, which significantly increases the charge density under ionic interactions, leading to an increase in electrostatic repulsion, causing a rise in swelling and an increase in the average size.

#### 3.1.4. FTIR Spectra and UV-Vis Spectroscopy

**Figure 6:**
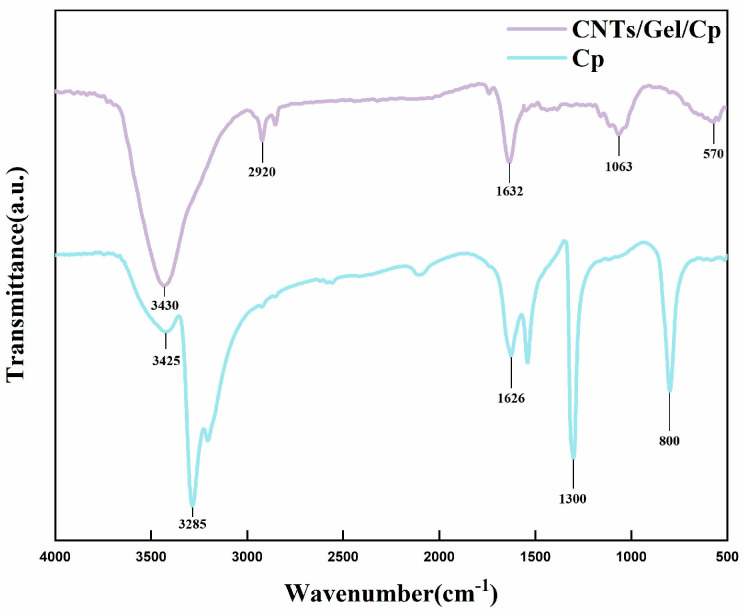
FTIR of Cp and CNTs/Gel/Cp. FTIR spectra of Cp show three peaks at 3285, 1300, and 800 cm^−1^. FTIR spectra of CNTs/Gel/Cp show two peaks at 3430 and 2920 cm^−1^.

Using infrared spectroscopy analysis, the chemical functional groups and their relative concentrations in Cp and CNTs/Gel/Cp were identified. Figure 6 shows three characteristic bands of Cp, located at 3285, 1300, and 800 cm^−1^, respectively. These bands are attributed to the absorption of N-H stretching vibrations in Cp, a characteristic of cisplatin [49]. The addition of functionalized carbon nanotubes, dopamine, and gelatin affected the optical characteristics of CNTs/Gel/Cp. Thus, the absorption spectra of the CNTs/Gel/Cp shifted to the left, compared to free Cp. The characteristic peak at 3430 cm^−1^ in the CNTs/Gel/Cp spectrum indicates that -OH is derived from the Michael addition reaction between -COOH and gelatin. However, the band widens because of the hydrogen bonding between cisplatin and CNTs/Gel. The presence of CH_2_ groups in CNTs/Gel/Cp increases the peak intensity in the 2920 cm^−1^ region [26]. Compared to Cp, CNTs/Gel/Cp showed an upward shift in the 500–1600 cm^−1^ region, indicating successful adsorption of cisplatin onto carbon nanotubes. Similar changes in optical properties were also observed by Mejri, A. et al. [42] in their study of cisplatin-loaded carbon nanotubes. These results suggest that Cp can bind to carbon nanotubes without altering either’s basic structure or function.

**Figure 7:**
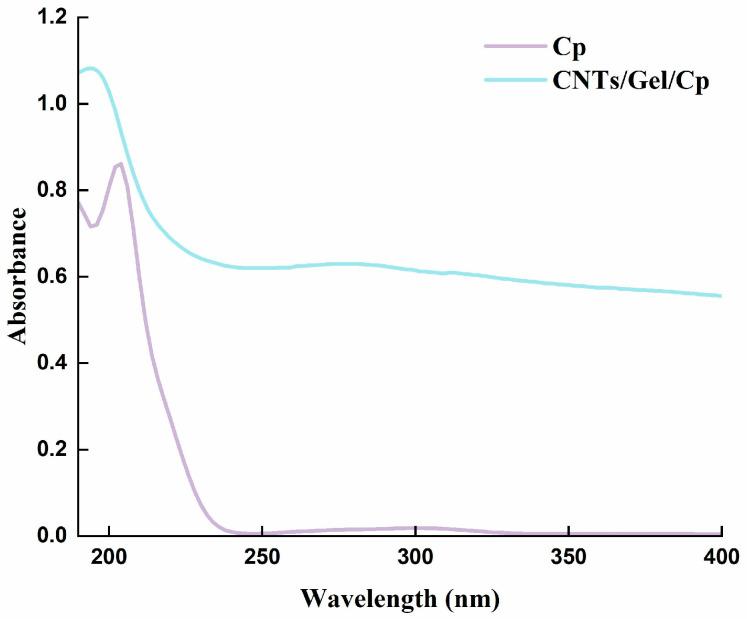
UV-vis of Cp and CNTs/Gel/Cp. A maximum peak for Cp at a wavelength of 204 nm, while CNTs/Gel/Cp showed a maximum peak at 194 nm with a slight leftward shift.

UV-visible (UV-vis) spectroscopy analysis was used to characterize the structure of functionalized carbon nanotubes and cisplatin, illuminating their chemical composition by analyzing the samples’ ultraviolet absorption. The results indicate a maximum peak for Cp at a wavelength of 204 nm, consistent with the reported findings in the literature [48], while CNTs/Gel/Cp showed a maximum peak at 194 nm with a slight leftward shift compared to Cp’s spectral peak, remaining broadly similar overall (Figure 7). These results imply that cisplatin has successfully bound to carbon nanotube surfaces.

#### 3.1.5. XRD Analysis

**Figure 8:**
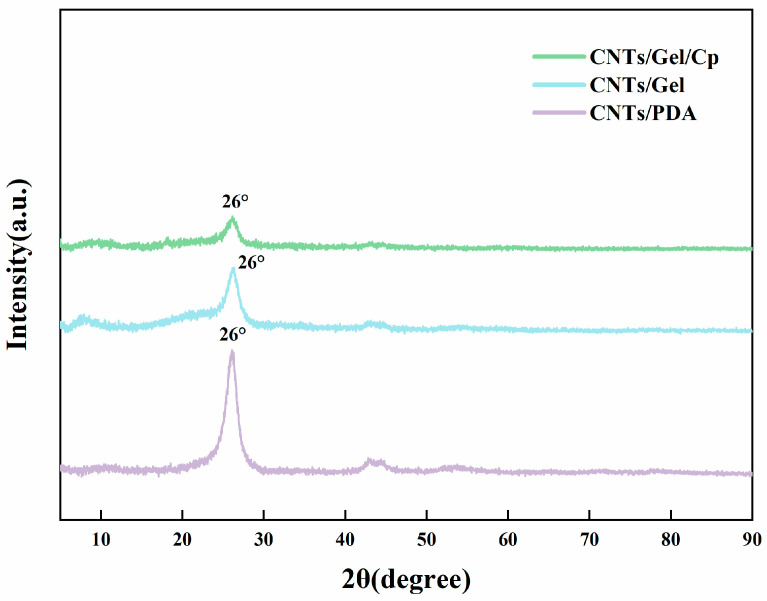
The XRD patterns of CNTs/PDA, CNTs/Gel, and CNTs/Gel/Cp. A peak was observed at 2θ = 26° in the XRD pattern of CNTs/PDA, which was weakened in the patterns of CNTs/Gel and CNTs/Gel/Cp.

The XRD spectra of CNTs/PDA, CNT/Gel, and CNTs/Gel/Cp are displayed in Figure 8. In the XRD pattern of CNTs/PDA, the peak (002) at 2θ = 26° could be attributed to the graphitic carbon of the hexagonal graphite structure (96-900-0047) [50], while it was diminished in the spectra of CNTs/Gel and CNTs/Gel/Cp. The aforementioned results indicated that the fundamental physical structure of carbon nanotubes remained largely unaltered during the surface modification process.

#### 3.1.6. Raman Spectroscopy

**Figure 9:**
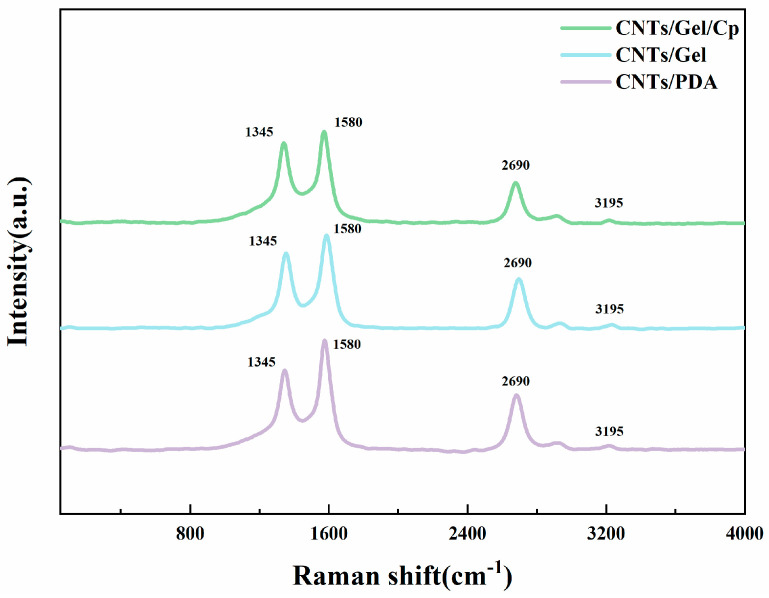
The Raman spectra of CNTs/PDA, CNTs/Gel, CNTs/Gel/Cp, and Cp were obtained. The D band was observed at 1345 cm^−1^ for all CNT samples, while the G band was observed at 1580 cm^−1^. D* and G* were the frequency doubling modes for the D and G peaks located at 2690 and 3195 cm^−1^, respectively.

Raman spectroscopy (Figure 9) served as a non-destructive means to characterize the CNTs and their derivatives. In Figure 9, the G and D peaks of CNTs were observed at 1580 and 1345 cm^−1^, respectively, indicating that no substantial structural damage occurred during the modification process, which occurred via physical adsorption on the surface of the carbon nanotubes. D* and G* were the frequency doubling modes for the D and G peaks located at 2690 and 3195 cm^−1^, respectively [51,52].

#### 3.1.7. TGA Analysis

**Figure 10:**
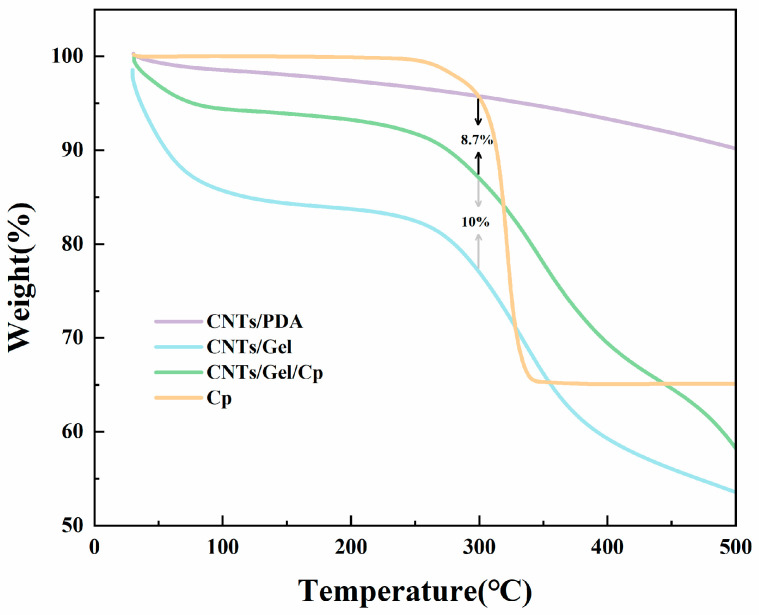
The TGA curves of CNTs/PDA, CNTs/Gel, CNTs/Gel/Cp, and Cp. CNTs/PDA, CNTs/Gel, and CNTs/Gel/Cp all underwent rapid weight loss when heated above 250 °C. Cp showed signs of decomposition when heated to 300 °C and completely decomposed at around 400 °C. CNTs/Gel experienced a weight loss of approximately 10% more than CNTs/Gel/Cp. CNTs/Gel experienced a much larger weight loss, approximately 18.7%, compared to CNTs/PDA. There was an approximate 8.7% difference in weight loss between CNTs/Gel/Cp and CNTs/PDA.

In order to further investigate the degree of functionalization and thermal stability of CNTs, the weight losses of CNTs/PDA, CNTs/Gel, CNTs/Gel/Cp, and Cp were measured by TGA (Figure 10). The organic or polymer substances present in CNTs/PDA, CNTs/Gel, and CNTs/Gel/Cp had shown rapid weight loss when above 250 °C. The thermal loss of CNTs/PDA at 500 °C was about 10.2% of the total weight, primarily due to the thermal decomposition of polydopamine.

Cp was observed to start decomposing at 300 °C and was fully decomposed at around 400 °C. Therefore, we selected the data point of 300 °C to compare the weight loss between different samples. As shown in Figure 10, the weight loss of the CNTs/Gel had been approximately 10% greater than that of CNTs/Gel/Cp, mainly because Cp had a higher decomposition temperature. When the gelatin had been grafted onto the surface of CNT/PDA, the organic content increased, resulting in a greater weight loss of CNT/Gel, approximately 18.7% more than that of CNT/PDA. The difference in weight loss between CNTs/Gel/Cp and CNT/PDA had been approximately 8.7%, mainly due to the decomposition of gelatin.

All the TGA curves confirmed that polydopamine and gelatin had been successfully coated onto the surface of CNTs, and cisplatin had been successfully loaded onto its surface. These results were consistent with our earlier findings [41].

### 3.2. The Loading Rate of Drugs

Drug loading and encapsulation efficiency are two common parameters in nano-pharmaceutical drug delivery systems [15]. The effectiveness of nano-drug delivery can be determined, as well as the efficacy of the drug’s encapsulation throughout the nanoparticle manufacturing process, by measuring Cp’s loading and encapsulation efficiency in CNTs/Gel/Cp at various weight ratios using ultraviolet-visible spectrophotometry.

Initially, when the mass ratio of Cp and CNTs/Gel was 1:2, 1:1, and 2:1, the loading rates were determined for three groups, respectively, with average rates of 2.65 ± 0.99, 10.83 ± 4.14, and 8.70 ± 2.78% obtained. The highest load rate of M_Cp_:M_CNTs/Gel_ = 1:1 among them (*p* < 0.05) indicated that the CNTs/Gel/Cp in this group had the best drug delivery effect (Table 2).

Subsequently, when the mass ratio of Cp to CNTs/Gel was 1:2, 1:1, and 2:1, the encapsulation rates of the three groups were determined, respectively, with average encapsulation rates of 7.94 ± 2.97, 21.67 ± 8.27, and 13.06 ± 4.17% obtained. The encapsulation rate of M_Cp_:M_CNTs/Gel_ = 1:1 was the highest (*p* < 0.05), indicating that CNTs/Gel/Cp in this group had the highest drug encapsulation efficiency during the preparation process (Table 2).

### 3.3. The Release of Cisplatin

**Figure 11:**
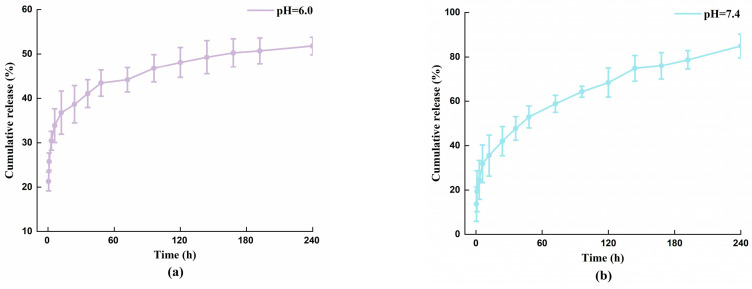
(**a**) The release of Cp at pH = 6.0. (**b**) The release of Cp at pH = 7.4. Under the pH = 6.0 condition, cisplatin’s initial release rate reached 30% within the first 3 h, and 39 and 43% were released at 24 and 48 h, respectively. Under the pH = 7.4 condition, the 24 and 48 h release rates were 42 and 53%, respectively. The results above indicate that, under weakly acidic conditions within the tumor (pH = 6.0), Cp can still be effectively released while maintaining a steady rate.

The tumor microenvironment is weakly acidic, and an ideal drug delivery system would respond to the acidic environment [15,20]. We studied the in vitro release behavior of the CNTs/Gel/Cp drug delivery system using the dialysis method to demonstrate its pH-responsive activity [26]. Under the conditions of pH = 7.4 and pH = 6.0, the cumulative release rate was calculated based on two standard curves (Cp-PBS_pH_ = 7.4 and Cp-PBS_pH_ = 6.0), and the Pt release curves of the two drug transport systems are shown in Figure 11a,b.

By observing the release rates at various intervals, CNTs/Gel/Cp exhibited comparable trends in two pH states. Under the pH = 6.0 condition (Figure 11a), cisplatin’s initial release rate reached 30% within the first 3 h, and 39 and 43% were released at 24 and 48 h, respectively. As time progressed, Cp continued to release at a steady rate of 1% per day, accumulating up to a release of 56% at 14 days (*p* < 0.05). Under the pH = 7.4 condition (Figure 11b), the 24 and 48 h release rates were 42 and 53%, respectively. With time, Cp continued to release at a constant rate of 3–5% daily. The cumulative release of Cp at 10 days was 85% (*p* < 0.05). In contrast, Cp’s cumulative release rate slightly decreased under the pH = 6.0 setting while maintaining a long-term, consistent release to produce a sustained-release effect. Overall, the drug release behavior is satisfactory and comparable to that of other studies [53].

The results above indicate that, under weakly acidic conditions within the tumor (pH = 6.0), cisplatin can still be effectively released while maintaining a steady rate. However, the release rate of cisplatin is, to some extent, affected by pH. The drug releases more persistently in acidic environments because a stronger non-bonded connection with gelatin-modified carbon nanotubes is formed. This fortified liaison yields an extended tenure of drug delivery superior to that observed under neutral circumstances. This finding is in line with what has been previously reported in the literature [15]. Compared to the pharmacokinetics of free drugs, the carbon nanotube drug delivery system loaded with Cp demonstrates pH responsiveness, and CNTs/Gel/Cp greatly enhances the amount of Cp released. The maximum drug release property of the pH-responsive nanocarrier lies in the ability to achieve intelligent drug release by adjusting the pH of the medium, which provides a possibility for the targeted delivery and treatment of CNTs/Gel/Cp, among other applications. In addition, it was proposed that the control of the release behavior of Cp trapped inside nanotubes could only be achieved by managing the sizes of the exit points, which include open ends and sidewall defects of the nanotubes [47,54]. Therefore, obstructing the exits of Cp further to achieve a pH-sensitive release curve might be a promising avenue for future investigation in carbon nanotube drug delivery systems. Moreover, the rationality of cellular uptake needs to be verified, and the efficacy of drug release at the cellular level and its subsequent absorption requires further elucidation.

### 3.4. In Vitro Antitumor Effect

**Figure 12:**
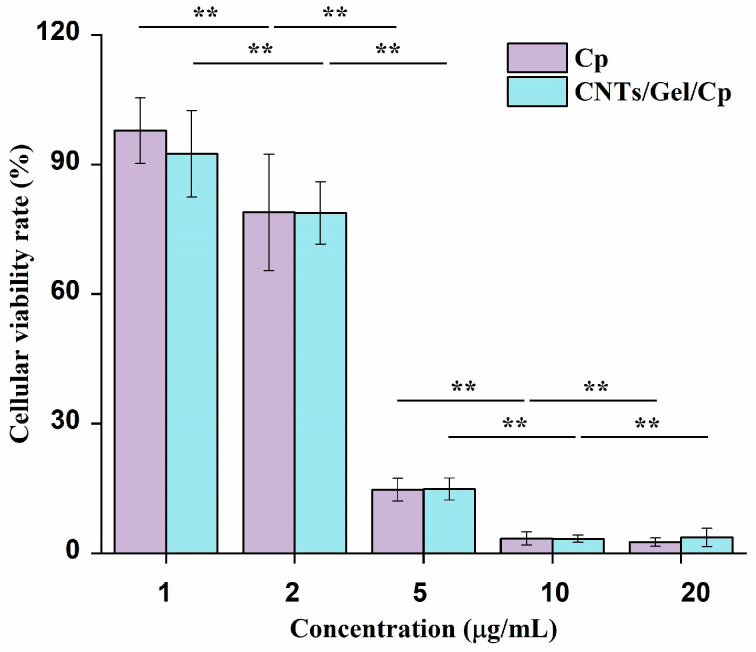
Human tongue squamous cell carcinoma Cal-27 cultured in suspensions of Cp and CNTs/Gel/Cp at different concentrations. Both groups had similar antitumor effects within 24 h. CNTs/Gel/Cp display antitumor properties that are comparable to cisplatin in a manner that varies with the dosage administered. Statistical analysis was performed by one-way ANOVA. The error bars represent the mean ± SD for n = 6, ** *p* < 0.01.

Using the CCK-8 method, the in vitro antitumor effect of the CNTs/Gel/Cp nanocarrier system was assessed against Cal-27 squamous cell carcinoma cells from the human tongue. This was also compared with the in vitro antitumor effect of Cp. In the experiment on cell viability of Cal-27 cells, both the CNTs/Gel/Cp group and Cp group were administered with drug concentrations of 1, 2, 5, 10, and 20 μg/mL. The CCK-8 test displayed the curve of cell activity, as shown in Figure 12, and revealed that both groups had similar antitumor effects within 24 h. Moreover, CNTs/Gel/Cp still exhibited antitumor effects like that of Cp in low concentrations (1 and 2 μg/mL) and high concentrations (5, 10, and 20 μg/mL) (*p* > 0.05). After 24 h of treatment, the antitumor effect of the CNTs/Gel/Cp nanocarrier system rose with the concentration increase (*p* < 0.05). At a 1 μg/mL concentration, the cell survival rate was around 92%, while at a 20 μg/mL concentration, the cell survival rate was only about 3%. Similar to this, the survival rate of the cells in the Cp group is roughly 97% at a concentration of 1 μg/mL but only about 2% at a concentration of 20 μg/mL (*p* < 0.05). Similar outcomes were also noted in earlier scholarly literature [53]. The results of the in vitro antitumor experiments showed that the CNTs/Gel/Cp nanocarrier system exhibits antitumor effects like that of Cp, indicating that the drug delivery system can effectively release the drug and achieve the purpose of killing tumor cells.

**Figure 13:**
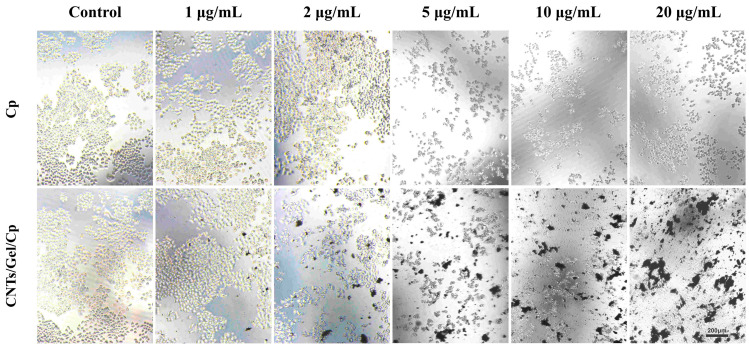
Human tongue squamous cell carcinoma Cal-27 cells incubated with different concentrations of Cp and CNTs/Gel/Cp. In the control group, cancer cells were observed to be densely clustered, and there was little difference in the number of cancer cells between the 1 μg/mL concentration group and the control group. The number of survived squamous carcinoma cells decreased as the concentrations of the Cp and CNTs/Gel/Cp groups increased (≥5 μg/mL).

Figure 13 depicts our observations of the extracellular shape, size, number, and distribution of Cal-27 human tongue squamous cell carcinoma cells under a microscope (100 × 100 μm). In the control group, cancer cells were observed to be densely clustered, and there was little difference in the number of cancer cells between the 1 μg/mL concentration group and the control group. This could mean that Cp and CNTs/Gel/Cp did not have a substantial antitumor impact at this concentration. At a concentration of 2 μg/mL, black CNTs/Gel/Cp particles were visible, and cancer cells were seen to be clustered together with fewer cancer cells in the CNTs/Gel/Cp group. The number of survived squamous carcinoma cells decreased as the concentrations of the Cp and CNTs/Gel/Cp groups increased (≥5 μg/mL). Moreover, the difference in the number of survived cells between the two groups was insignificant. The cancer cell distribution was relatively scattered, indicating similar antitumor cell effects between the two groups. These experimental findings agree with other researchers who have discovered that nanoparticles coated with cisplatin can have potent antitumor effects [55].

**Figure 14:**
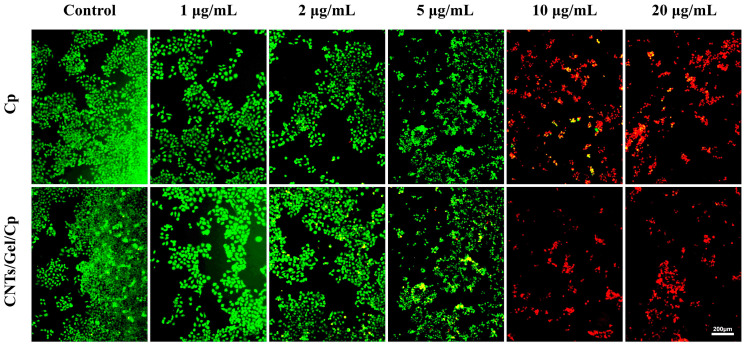
AO/EB staining assay of Cal-27 cells incubating with Cp and CNTs/Gel/Cp in different concentrations. The green fluorescence indicates live cancer cells, while the red fluorescence indicates cancer cells that have perished. At concentrations of 1 and 2 μg/mL for both CNTs/Gel/Cp and Cp groups, no significant differences in cellular morphology were observed between the two groups. At concentrations of 10 and 20 μg/mL, both groups exhibited a significant increase in red fluorescence, indicating a marked increase in cancer cell death.

In order to visually demonstrate the antitumor effect of CNTs/Gel/Cp, we employed the AO/EB staining experiment to investigate the impact of the nano-drug delivery system on cell apoptosis and survival (Figure 14). At concentrations of 1 and 2 μg/mL for the CNTs/Gel/Cp and Cp groups, a higher concentration of cancer cells was observed, with most of them being alive (green fluorescence) and very few dead cancer cells (red fluorescence), with no significant difference in cellular morphology between the two groups. There was no clear red fluorescence at 5 μg/mL. However, there was a noticeable change in the cancer cell morphology, showing that the concentration boosted the antitumor action of the CNTs/Gel/Cp and Cp to some extent. At concentrations of 10 and 20 μg/mL, a significant increase in red fluorescence was observed, indicating a marked increase in cancer cell death, and the CNTs/Gel/Cp and Cp groups showed a strong antitumor effect [56].

## 4. Conclusions

Based on our early study, we utilized gelatin-modified carbon nanotubes (CNTs/Gel) to load cisplatin (Cp) and developed a pH-responsive nano-drug delivery system (CNTs/Gel/Cp), enabling the precise and gradual release of medication. The best drug-loading rate and encapsulation efficiency occur when the Cp and CNTs/Gel mass ratio is 1:1, leading to exceptional drug-loading effectiveness and greater drug encapsulation efficiency in the nanomedicine delivery system. The CCK-8 assay and AO/EB staining results from the in vitro antitumor investigations show that the CNTs/Gel/Cp nanocarrier system has antitumor effects comparable to Cp. In summary, the CNTs/Gel/Cp nano-drug delivery system demonstrates a precise and slow drug release, which effectively exhibits good antitumor effects in cancer treatment. It is important to note, however, that this study has limitations. For instance, the tumor acidic microenvironment was only simulated in vitro, and further research and exploration on the release of CNTs/Gel/Cp and their antitumor effects in the in vivo tumor microenvironment needs to be conducted by constructing animal models. Therefore, the CNTs/Gel/Cp nano-drug delivery system is a reference point for the clinical application of modified CNTs as an emerging drug delivery technology. It provides theoretical and experimental evidence for a promising cancer treatment.

## Figures and Tables

**Table 1:** The percentage of element contents (%) of each group.

Group	N Element	Cl Element	Pt Element
CNTs/Gel	98.70%	1.30%	0.00%
CNTs/Gel/Cp	62.20%	10.81%	26.99%

**Table 2:** Loading and encapsulation rates (n = 3, x ± s).

M_Cp_:M_CNTs/Gel_	Loading Rates	Encapsulation Rates
1:2	2.65 ± 0.99 ^a^	7.94 ± 2.97 ^c^
1:1	10.83 ± 4.14 ^b^	21.67 ± 8.27 ^d^
2:1	8.70 ± 2.78 ^b^	13.06 ± 4.17 ^c^

The groups identified by the same lowercase letter down the column are not statistically different (*p* > 0.05).

## Data Availability

No new data were created or analyzed in this study.
